# Diferenças entre os Sexos nos Desfechos de Pacientes com Infarto do Miocárdio com Supradesnivelamento do Segmento ST Submetidos à Intervenção Coronária Percutânea Primária

**DOI:** 10.36660/abc.20220673

**Published:** 2023-06-01

**Authors:** Victoria B. Milan, Yasmin F. S. Alves, Guilherme P. Machado, Gustavo Neves de Araujo, Ana Maria Krepsky, Angelo Chies, Matheus Niches, Julia Fracasso, Sandro Cadaval Goncalves, Marco Wainstein, Carisi Anne Polanczyk

**Affiliations:** 1 Universidade Federal de Ciências da Saúde de Porto Alegre Faculdade de Medicina Porto Alegre RS Brasil Universidade Federal de Ciências da Saúde de Porto Alegre Faculdade de Medicina, Porto Alegre, RS – Brasil; 2 Hospital de Clínicas de Porto Alegre Porto Alegre RS Brasil Hospital de Clínicas de Porto Alegre, Porto Alegre, RS – Brasil; 3 Programa de Pós-Graduação em Cardiologia e Ciências Cardiovasculares Universidade Federal do Rio Grande do Sul Porto Alegre RS Brasil Programa de Pós-Graduação em Cardiologia e Ciências Cardiovasculares, Universidade Federal do Rio Grande do Sul, Porto Alegre, RS – Brasil; 4 Instituto de Cardiologia de Santa Catarina São José SC Brasil Instituto de Cardiologia de Santa Catarina, São José, SC – Brasil; 5 Imperial Hospital de Caridade Florianópolis SC Brasil Imperial Hospital de Caridade, Florianópolis, SC – Brasil; 6 Universidade Federal do Rio Grande do Sul Porto Alegre RS Brasil Universidade Federal do Rio Grande do Sul, Porto Alegre, RS – Brasil

**Keywords:** Infarto do Miocárdio com Supradesnivelamento do Segmento ST (IAMCSST, Características Sexuais, Idoso, Intervenções Coronárias Percutâneas

## Abstract

**Fundamento:**

Vários estudos têm mostrado que as mulheres não recebem tratamento adequado e apresentam piores desfechos após infarto agudo do miocárdio com supradesnivelamento do segmento ST (IAMCSST). Por isso, é necessário investigar questões relacionadas ao gênero para melhor lidar com esse problema no Brasil.

**Objetivo:**

Determinar se existe associação entre o sexo feminino e eventos adversos em uma coorte contemporânea de pacientes com IAMCSST submetidos à intervenção coronária percutânea primária (ICPp).

**Métodos:**

Este foi um estudo prospectivo do tipo coorte de pacientes com IAMCSST submetidos à ICPp em um hospital universitário terciário entre março de 2011 e dezembro de 2021. Os pacientes foram categorizados em grupos de acordo com o sexo ao nascimento. O primeiro desfecho clínico foi ECAM em longo prazo. Os pacientes foram acompanhados por um período máximo de cinco anos. Um nível de significância bilateral de 0,05 foi aplicado em todos os testes de hipóteses.

**Resultados:**

Entre os 1457 pacientes internados por IAMCSST no período do estudo, 1362 foram incluídos e 468 (34,4%) eram do sexo feminino. As mulheres apresentaram maior prevalência de hipertensão (73% vs. 60%, p<0,001), diabetes (32% vs. 25%, p=0,003) e classe Killip 3-4 na internação (17% vs. 12%, p=0,01); o escore de risco TIMI foi maior nas mulheres [4 (2, 6) vs. 3 (2, 5), p<0.001]. A mortalidade hospitalar não foi diferente entre os grupos (12,8% vs. 10,5%; p=0,20). Os ECAMs foram numericamente maiores nas mulheres que nos homens tanto durante a internação (16,0% vs. 12,6%, p=0,085) como em longo prazo (28,7% vs. 24,4%, p=0,089), com significância limítrofe. Após a análise multivariada, o sexo feminino não foi associado a ECAMs (HR = 1,14; IC95% 0,86 – 1,51; p = 0,36).

**Conclusão:**

Em uma coorte prospectiva contemporânea de pacientes com IAMCSST submetidos à ICPp, pacientes do sexo feminino apresentaram idade mais avançada e mais comorbidades no basal que os pacientes do sexo masculino, mas não houve diferenças significativas entre os sexos quanto aos desfechos adversos no hospital ou em longo prazo.

## Introdução

A doença cardiovascular está entre as principais causas de morbimortalidade no mundo.^1^ A prevalência da doença arterial coronariana (DAC) tem aumentando entre homens e mulheres no Brasil, e já representa 13% das mortes na população geral.^2^ Historicamente, a DAC afeta mais homens que mulheres,^3^ sendo que os homens apresentam o primeiro episódio de infarto do miocárdio (IM) no mínimo sete anos antes que as mulheres.^3,4^ Contudo, estudos mostram que as mulheres, mesmo com menos eventos, apresentam piores desfechos após um infarto agudo do miocárdio (IAM), principalmente se apresentam supradesnivelamento do segmento ST (IAMCSST).^5,6^

Estudos têm descrito várias teorias socioambientais plausíveis para os piores desfechos entre as mulheres que apresentam IAM.^7^ Ainda, grande parte do conhecimento atual sobre as diferenças entre os sexos no manejo do IAMCSST baseia-se em estudos conduzidos em países de alta renda. Estatísticas mundiais mostram que as taxas de mortalidade cardiovascular nesses países caíram cerca de 10% nos últimos 20 anos, enquanto em países de renda média-baixa, como o Brasil, essas taxas aumentaram em cerca de 40%.^1^ Esses dados também confirmam a existência de diferenças significativas na expectativa de vida entre homens e mulheres de diferentes situações econômicas e geográficas.

Considerando que a intervenção coronária percutânea primária (ICPp) é o tratamento padrão em pacientes internados por IAMSST,^8^ este estudo prospectivo do tipo coorte tem como objetivo investigar a relação entre sexo e desfechos adversos em pacientes internados por IAMCSST submetidos à ICPp em um hospital terciário no sudeste do Brasil.

## Métodos

### Dados, delineamento e população do estudo

Este estudo prospectivo foi conduzido em um hospital universitário terciário do sudeste do Brasil entre março de 2011 e dezembro de 2021. Os pacientes elegíveis para inclusão foram adultos (≥18 anos de idade) consecutivos, com suspeita de IAMCSST, com base na presença de dor torácica típica em repouso associada à elevação do segmento ST ou anormalidades que preenchessem os critérios diagnósticos para IAMCSST de acordo com as diretrizes atuais.^8^ Os critérios de exclusão foram IAM sem supradesnivelamento do segmento ST (IAMSSST), IM com artérias coronárias não obstrutivas, e outros diagnósticos finais. Mais detalhes do nosso protocolo foram publicados em outro estudo.^9^Todos os participantes assinaram um termo de consentimento. Este estudo foi aprovado pelo comitê de ética em pesquisa da instituição. O artigo foi escrito seguindo as diretrizes STROBE para a redação de estudos observacionais.^10^

As amostras de sangue foram coletadas por punção venosa na internação para exames laboratoriais em geral. Todos os pacientes receberam tratamento otimizado de acordo com diretrizes atuais.^8^ As técnicas da ICP (como pré-dilatação, implantação direta do *stent*), foram conduzidas a critério do operador. Ecocardiografia foi realizada nas primeiras 48 horas de internação segundo rotina do hospital.

Dados dos prontuários médicos foram transferidos para formulários padronizados para relatos de caso. As seguintes variáveis foram coletadas: características basais, história clínica, características do procedimento, estratégia de reperfusão, tratamento farmacológico na unidade de terapia intensiva, necessidade de aparelhos de monitoramento hemodinâmico e terapias na alta. A classificação de Killip foi usada na primeira avaliação na admissão antes da revascularização coronária. Acompanhamento aos 30 dias e em longo prazo foi conduzido por visitas médicas e contato telefônico até um máximo de 60 meses. Dados do estudo foram transferidos e manipulados usando a ferramenta de captura de dados eletrônicos REDCap do Hospital de Clínicas de Porto Alegre. Os pacientes foram categorizados nos grupos de acordo com o sexo.

### Desfechos

O desfecho clínico primário foi eventos cardiovasculares adversos maiores (ECAMs) – desfecho composto por mortalidade por todas as causas, novo IM, acidente vascular cerebral, trombose de *stent* e revascularização do vaso alvo. O tratamento de lesões não culpadas não foi considerado como nova revascularização. O desfecho secundário foi a análise individual dos ECAMs. Novo IM foi definido de acordo com a definição universal mais recente de IM.^11^ Uma segunda análise foi realizada para mortalidade de subgrupos estratificados por idade. O acidente vascular cerebral foi definido como um déficit neurológico novo, focal, de início repentino, de causa presumivelmente cerebrovascular, irreversível (ou que resulte em óbito) e não causado por outras causas identificadas.

Desfechos dos procedimentos também foram descritos. Um procedimento bem-sucedido foi definido como um escore de TIMI 2 ou 3 e estenose residual < 30%. A ausência de refluxo foi definida como reperfusão miocárdica subótima por uma parte da circulação coronária sem evidência angiográfica de obstrução mecânica do vaso. Embolização distal foi definida como um defeito de enchimento distal com interrupção abrupta em um dos ramos da artéria coronária periférica do vaso relacionado ao infarto, distal ao local da angioplastia. Parada cardíaca foi definida como uma parada cardíaca que ocorreu durante o procedimento, com necessidade de procedimentos de ressuscitação (p.ex., ventilação, compressão torácica, desfibrilação).

### Análise estatística

As variáveis contínuas foram expressas em média ± desvio padrão (DP) ou mediana (intervalo interquartil), de acordo com a normalidade dos dados. A normalidade da distribuição de cada variável foi avaliada pelo teste de Shapiro-Wilk. As variáveis categóricas foram expressas como frequências relativas e absolutas. Os grupos dos pacientes foram comparados pelo teste t de *Student* para amostras independentes (para variáveis com distribuição normal) ou o teste de Mann-Whitney (para outras variáveis) quanto as variáveis contínuas e o teste do qui-quadrado ou o teste exato de Fisher quanto as variáveis categóricas. Para a análise multivariada, realizou-se análise de regressão de Cox para desfecho primário, com inclusão de variáveis clínicas importantes. Um modelo multivariado foi ajustado quanto ao sexo, idade, IM na parede anterior classe Killip 3 ou 4, hipertensão, diabetes, creatinina na admissão, doença de múltiplos vasos, uso prévio de ácido acetilsalicílico (AAS), IM prévio, uso prévio ou atual de medicamentos, tempo entre dor e atendimento, tabagismo, sucesso angiográfico, índice de massa corporal, bloqueio atrioventricular (AV) completo. Curvas de sobrevida de Kaplan-Meier foram construídas para apresentar dados de tempo para o evento não ajustado para os desfechos investigados e comparadas pelo teste de log rank usando o programa de estatística MedCalc versão 14.8.1 (MedCalc Software bvba, Oostende, Bélgica). As demais análises foram realizadas usando o programa SPSS para Windows, versão 26.0. (IBM Corp., Armonk, NY). Em todos os testes de hipóteses, aplicou-se um nível de significância bilateral de 0,05.

## Resultados

### Características clínicas basais

**Figura 1:**
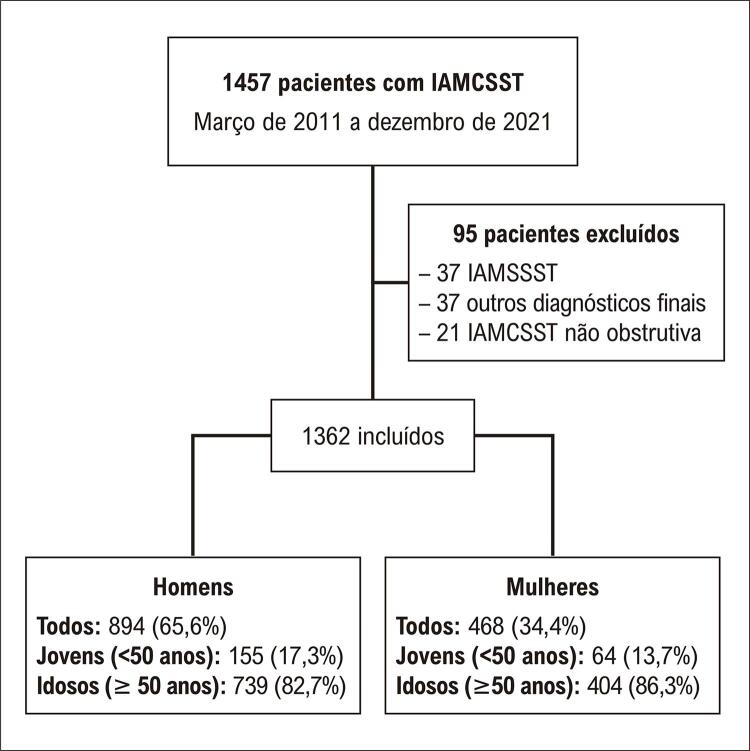
– Fluxograma da inclusão de pacientes. IAMCSST: infarto agudo do miocárdio com supradesnivelamento do segmento ST; IAMSSST: IAM sem supradesnivelamento do segmento ST

Dos 1457 pacientes admitidos com IAMCSST no período do estudo, 1362 (468 mulheres e 894 homens) foram incluídos na análise (Figura 1).

A idade média das mulheres foi 62,8 anos e dos homens 60,2 anos. Hipertensão, diabetes, classe Killip 3-4 na admissão e bloqueio AV completo foram mais frequentes e o escore de risco TIMI foi mais alto nas mulheres que nos homens. Os homens apresentaram fração de ejeção pós-IM mais baixa que as mulheres. Outras características basais dos pacientes, por sexo, estão resumidas na Tabela 1.

**Tabela 1:** – Características basais dos pacientes por sexo

	Feminino (n=468)	Masculino (n=894)	Valor p
Idade, anos	62,8 (±12,2)	60,2 (±11,5)	<0,0001
IMC, Kg/m^2^	26 (23,3-29,2)	26,7 (24,2-29,8)	0,01
Caucasiano	407 (87)	799 (89,6)	0,24
Hipertensão	342 (73,1)	538 (60,2)	<0,001
Diabetes mellitus	152 (32,5)	223 (24,9)	0,003
Terapia com insulina	54 (35,5)	56 (24,9)	0,02
História familiar de DAC	74 (16,2)	165 (18,9)	0,20
Consumo de álcool e / ou drogas	15 (3,2)	142 (16,0)	<0,0001
Fibrilação atrial	13 (2,8)	25 (2,8)	0,97
Tabagismo prévio ou atual	290 (62,0)	588 (65,8)	0,16
Dislipidemia	71 (15,2)	130 (14,6)	0,78
Uso de aspirina	87 (18,6)	148 (16,6)	0,36
IAM prévio	55 (11,8)	133 (14,9)	0,11
AVC prévio	47 (10,0)	68 (7,6)	0,12
CABG prévio	11 (2,4)	27 (3,0)	0,48
ICP prévia	52 (11,2)	104 (11,7)	0,77
DRC	19 (4,1)	46 (5,1)	0,37
Doença psiquiátrica	42 (9,0)	41 (4,6)	0,001
Horário comercial	242 (51,7)	508 (56,8)	0,13
Finais de semana	161 (34,4)	285 (31,9)	0,35
**Classe Killip**			0,003
I	284 (60,7)	630 (70,5)	
II	105 (22,4)	157 (17,6)	
III	21 (4,5)	26 (3,1)	
IV	58 (12,4)	79 (8,8)	
Classe Killip III-IV	79 (16,9)	107 (12,0)	0,01
Bloqueio AV completo	40 (8,5)	42 (4,7)	0,005
Parada cardíaca súbita	45 (9,6)	93 (10,4)	0,64
**Extensão da DAC**			0,06
1 vaso	176 (37,8)	298 (33,7)	
2 vasos	124 (26,7)	289 (32,7)	
Doença de múltiplos vasos	165 (35,5)	298 (33,7)	
Trombólise	17 (3,7)	38 (4,3)	0,59
Revascularização completa	121 (59,3)	221 (56,4)	0,49
Tempo dor-porta, min	300 (180-503)	300 (180-480)	0,24
Tempo porta-balão, min	70 (57-95)	73 (60-98)	0,35
Tempo de isquemia, min	365 (250-561)	370 (243 -548)	0,48
PAS, mmHg	130 (110-150)	130 (112-150)	0,48
FC, bpm	82 (70-94)	80 (69-93)	0,20
Escore de risco TIMI	4 (2-6)	3 (2-5)	<0,0001
Creatinina, mg/dL	0,82 (0,68-1,1)	1,01 (0,85-1,29)	<0,0001
Hemoglobina, g/dL	12,7 (11,6-13,7)	14,2 (13-15,1)	<0,0001
RNL	6,61 (3,4-9,2)	6,5 (4,1-10,0)	0,053
FEVE, %	53 (41-62)	48 (40-58)	<0,0001
**Características do procedimento**			
Contraste, ml	152 (120-206)	175 (140-230)	0,012
Radiação, mGy	1282 (806-2246)	1649 (1026-2543)	<0,0001
Total de *stents*	1 (1-2)	1 (1-2)	0,41
Comprimento do *stent*, mm	32 (22-51)	30 (23-48)	0,90

Valores expressos em média (DP, desvio padrão), mediana (intervalo interquartil) ou número (%); IMC: índice de massa corporal; IAM: infarto agudo do miocárdio; CABG: bypass da artéria coronária; ICP: intervenção coronária percutânea; DRC: doença renal crônica; AV: atrioventricular; DAC: doença arterial coronariana, PAS: pressão arterial sistólica; FC: frequência cardíaca; RNL: razão neutrófilo-linfócito; FEVE: fração de ejeção do ventrículo esquerdo.

### Desfechos

**Figura 2:**
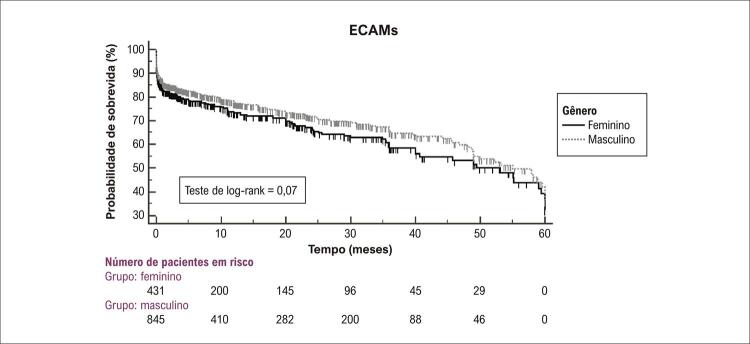
– Curvas tempo para o evento para o desfecho primário “eventos cardiovasculares adversos maiores (ECAMs)”; as taxas de evento foram calculadas pelo método Kaplan-Meier e comparadas pelo teste de log-rank.

A incidência de ECAMs em longo prazo (mediana de 41 meses) foi 31,4% nas mulheres e 26,5% nos homens [*hazard ratio* (HR) = 1,14; intervalo de confiança de 95% (IC 95%) = 0,86-1,51; p =0,36) (Figura 2 e Figura Central).

**Figura Central:**
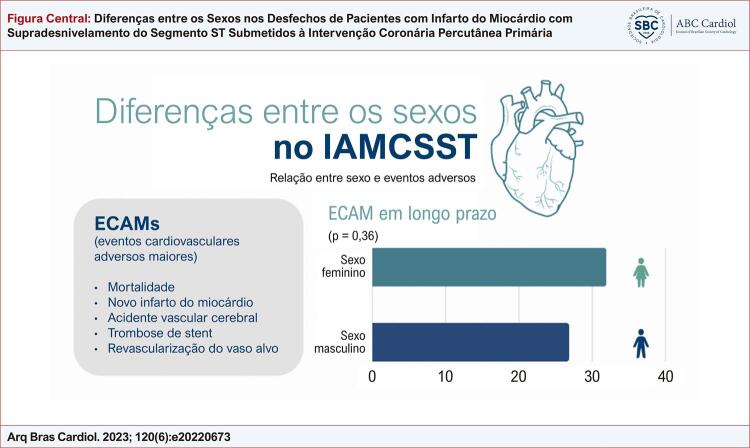
: Diferenças entre os Sexos nos Desfechos de Pacientes com Infarto do Miocárdio com Supradesnivelamento do Segmento ST Submetidos à Intervenção Coronária Percutânea Primária

**Figura 3:**
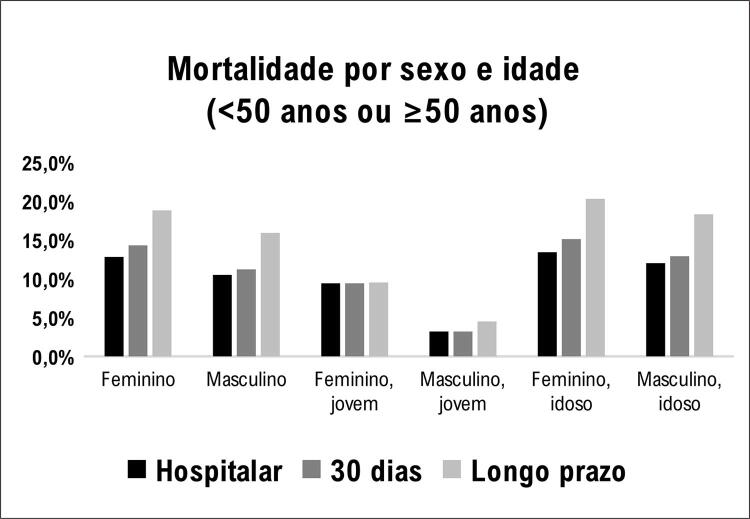
– Incidência de mortalidade por sexo e idade.

No geral, a mortalidade hospitalar foi 11,3%, sem diferença entre homens e mulheres (Figura 3).

Revascularização incompleta na alta hospitalar foi associada com maior mortalidade nos pacientes do sexo masculino (14 vs. 6,3%, p=0,01), mas não nos pacientes do sexo feminino (10,7 vs. 12%, p=0,82). Não foram observadas diferenças na ocorrência de IM, acidente vascular cerebral e ECAMs durante a internação ou na ocorrência de IM, acidente vascular cerebral, e revascularização do vaso alvo em longo prazo entre os grupos (Tabela 3).

**Tabela 3:** – Distribuição dos eventos adversos de acordo com o sexo

	Feminino (468)	Masculino (894)	Valor p
**Desfecho primário**			
ECAM longo prazo	148 (31,7)	238 (26,7)	0,36
**Desfechos secundários**			
Morte hospitalar	60 (12,8)	94 (10,5)	0,20
IAM durante a internação	10 (2,1)	14 (1,6)	0,44
Trombose de *stent* durante a internação	5 (1,1)	10 (1,1)	0,93
AVC durante a internação	10 (2,1)	11 (1,2)	0,19
ECAM durante a internação	75 (16,0)	113 (12,6)	0,08
**Longo prazo**			
Morte	88 (18,8)	142 (15,9)	0,17
IAM	38 (8,2)	58 (6,5)	0,25
AVC	23 (4,9)	26 (2,9)	0,05
Trombose de *stent*	22 (4,7)	38 (4,3)	0,70
Revascularização de vaso alvo	34 (7,3)	52 (5,8)	0,29

IAM: infarto agudo do miocárdio; ECAM: eventos cardiovasculares adversos maiores; AVC: acidente vascular cerebral.

Os medicamentos na alta hospitalar foram diferentes entre os pacientes do sexo masculino e feminino. Enquanto bloqueadores de receptor de aldosterona e bloqueadores de canal de cálcio eram mais comumente prescritos às pacientes de sexo feminino, os inibidores de ECA eram mais comuns na lista de alta dos pacientes do sexo masculino. Não foram observadas diferenças quanto às demais medicações (Tabela 4).

**Tabela 4:** – Medicamentos prescritos aos pacientes na alta hospitalar, por sexo

	Feminino (n=486)	Masculino (n=894)	Valor p
Aspirina	381 (93,4)	755 (94,4)	0,49
Clopidogrel	374 (91,7)	739 (92,4)	0,65
Estatinas	368 (90,2)	729 (91,1)	0,59
Betabloqueador	355 (87,0)	710 (88,8)	0,37
Outros agentes antiplaquetários	5 (1,2)	12 (1,6)	0,58
BRA	27 (6,6)	29 (3,6)	0,01
IECA	312 (76,5)	660 (82,5)	0,01
Espironolactona	27 (6,6)	66 (8,3)	0,31
Digoxina	11 (2,7)	20 (2,5)	0,83
Varfarina	18 (4,4)	42 (5,3)	0,52
NOAC	7 (1,7)	28 (3,5)	0,08
Bloqueador de canal de cálcio	16 (3,9)	16 (2,0)	0,04

BRA: bloqueador de receptor de angiotensina; IECA: inibidores de enzima conversora de angiotensina; NOAC: anticoagulantes orais não antagonistas da vitamina K.

### Análise multivariada

Idade, Killip 3-4 na internação, doença de múltiplos vasos, uso anterior de AAS, e FEVE foram preditores independentes de ECAMs na população geral. Creatinina e FEVE foram preditores independentes de ECAMs nas mulheres, mas não nos homens, e IM na parede anterior foi preditor de ECAM nos homens e não nas mulheres (Tabela 5).

**Tabela 5:** – Preditores de Eventos Cardiovasculares Adversos Maiores (ECAMs) nos pacientes de acordo com o sexo

	Total				Feminino				Masculino			
		
	HR	IC95%		Valor p	HR	IC95%		Valor p	HR	IC95%		Valor p
Feminino	1,14	0,86	1,51	0,36								
Idade	1,02	1,00	1,03	0,003	1,00	0,98	1,02	0,66	1,16	0,82	1,64	0,38
IM na parede anterior	1,09	0,83	1,44	0,52	0,72	0,44	1,19	0,20	1,92	1,19	3,08	<0,01
Killip 3 ou 4	2,15	1,53	3,03	<0,01	1,63	0,92	2,89	0,09	0,86	0,61	1,23	0,43
Creatinina	1,10	0,99	1,22	0,05	1,57	1,23	1,99	<0,01	1,31	0,88	1,93	0,17
Doença de múltiplos vasos	1,65	1,21	2,26	<0,01	2,66	1,51	4,67	<0,01	1,73	1,12	2,67	0,01
Uso prévio de AAS	1,59	1,14	2,23	0,01	1,01	0,57	1,78	0,96	0,89	0,54	1,46	0,65
FEVE	0,98	0,97	0,99	0,01	0,97	0,95	0,99	<0,01	0,71	0,39	1,28	0,26

AAS: ácido acetilsalicílico; FEVE: fração de ejeção do ventrículo esquerdo; IC: intervalo de confiança; IM: infarto do miocárdio.

O modelo foi ajustado por sexo, idade, IM na parede anterior, Killip classe 3 ou 4, hipertensão, diabetes, creatinina na internação, doença de múltiplos vasos, uso prévio de AAS, IN prévio, medicamentos atuais e anteriores, tempo entre o sintoma e o atendimento (dor-porta), tabagismo, FEVE, sucesso angiográfico, índice de massa corporal, e bloqueio AV completo.

## Discussão

Em uma coorte prospectiva de pacientes com IAMCSST, não encontramos diferenças significativas entre o sexo masculino e feminino quanto à mortalidade e ECAMs. Pacientes do sexo feminino apresentaram mais características de alto risco no basal, e maior número de desfechos adversos durante a internação e em longo prazo, embora as diferenças foram parcialmente neutralizadas após a análise multivariada. Finalmente, as mulheres eram menos propensas a receberem alta hospitalar com terapia medicamentosa baseada em diretrizes. Este estudo reproduz achados de estudos anteriores e contribui para os dados escassos sobre diferenças entre sexos no manejo de IAMCSST em países em desenvolvimento.

Similar a aparentemente todos os estudos sobre IAMCSST publicados, a prevalência de pacientes do sexo masculino foi maior em nossa coorte, o que pode ser explicado por dois aspectos. Primeiro, os homens apresentam uma maior prevalência de síndromes coronarianas agudas (2,3% vs. 1,2%),^2^ e a presente análise mostrou que a prevalência de IAM prévio foi maior nos homens. Segundo, o IAMCSST é subdiagnosticado nas mulheres devido à presença de sintomas atípicos e ao fato dessas pacientes terem menor acesso à ICPp, com vários estudos indicando que os homens apresentam uma probabilidade duas vezes maior de serem submetidos à terapia de reperfusão que as mulheres.^12-15^

As diferenças no perfil demográfico entre os sexos também foram similares a de estudos anteriores, em que as mulheres são geralmente mais velhas e apresentam mais comorbidades.^13-15^ Em relação ao fator idade, o estrogênio parece ter um efeito protetor,^7,15^ e as mulheres parecem ter maior adesão à prevenção primária.^3^ Uma análise prévia com 1,2 milhões de pacientes com IAMCSST revelou maior mortalidade em mulheres jovens com idade entre 19 e 49 anos, em um modelo ajustado por terapia de reperfusão (3,9% vs. 2,6%, p=0,003), o que, contudo, não foi confirmado em nosso estudo.^14^ As comorbidades exercem diferentes pesos na patogênese do IAM entre os sexos, com maior impacto sobre as mulheres. O diabetes mellitus, por exemplo, aumenta as chances de IAM em até três vezes nas mulheres em comparação aos homens.^12^ Em nossa análise, variáveis diferentes foram preditoras independentes de ECAMs em longo prazo nas mulheres (creatinina e FEVE) e nos homens (IAM na parede anterior).

Em nosso estudo, diferenças basais significativas entre os sexos não foram traduzidas em diferenças estatisticamente significativas na mortalidade e ECAMs durante a internação hospitalar. No entanto, estudos com diferentes populações têm apresentado resultados conflitantes. Um estudo avaliando 2,8 milhões de pacientes com IAMCSST submetidos à ICPp mostrou taxas de mortalidade e de complicações vasculares significativamente mais altas entre as mulheres.^16^ Pesquisadores espanhóis analisaram uma mostra de 680 indivíduos nonagenários, com incidência de 45% de IAMCSST e 35% de ICPp, e concluíram que as taxas de mortalidade hospitalar não foram diferentes entre homens e mulheres (16% vs. 18%; p=0,4).^16^ Em um estudo australiano, os autores investigaram o status anterior e posterior ao procedimento de revascularização nos pacientes com IAMCSST submetidos à ICP, e concluíram que a mortalidade nas mulheres foi fortemente associado com revascularização incompleta.^6^ Na presente análise, esse resultado foi observado nos pacientes do sexo masculino.

Tratamentos de doenças cardiovasculares baseados em diretrizes atuais baseiam-se em dados obtidos de pacientes predominantemente do sexo masculino.^12^ Recentemente, várias inciativas têm proposto uma maior atenção em se atingir igualdade de sexos na ciência cardiovascular com o objetivo de melhorar nosso entendimento sobre a fisiopatologia da doença cardiovascular e seu impacto nos desfechos adversos.^17,18^ Assim, estudos sobre essas questões em diferentes cenários continuam importantes para melhorar nossa prática clínica diária.

Este estudo tem limitações que são inerentes a estudos observacionais. Alguns dados foram obtidos retrospectivamente e outros por contato telefônico, o que pode resultar em informações menos confiáveis. Além disso, limitações existem devido ao tamanho amostral relativamente pequeno em comparação a estudos com populações maiores e período mais curto de acompanhamento. Este foi um estudo unicêntrico no sudeste do Brasil e pode não ser representativo para todo o país que apresenta diferenças culturais significativas entre as regiões. Ainda, encontramos uma alta taxa de mortalidade em nossa amostra, o que pode ser justificada pela gravidade da doença de nossos pacientes (aproximadamente 13% apresentaram classe Killip III/IV), possivelmente por apresentação tardia no hospital, o que representa um risco basal muito alto. Contudo, o presente estudo é um registro de pacientes consecutivos, não selecionados, de um hospital terciário de referência para tratamento de síndromes coronarianas agudas, de modo que os dados são altamente aplicáveis na prática clínica diária.

## Conclusão

Em uma coorte prospectiva contemporânea de pacientes com IAMCSST submetidos à ICPp, as pacientes do sexo feminino eram mais velhas e apresentaram mais comorbidades no basal, sem diferenças significativas em termos de desfechos hospitalares no hospital e em longo prazo. Infelizmente, as mulheres apresentaram menor probabilidade que os homens de receberem alta hospitalar com terapia medicamentosa baseada em diretrizes. Nós esperamos que essa informação ajude médicos a proporcionarem melhor tratamento a esse grupo de pacientes em diferentes contextos sociais.
