# Supplementary material for: Diferenças entre os Sexos nos Desfechos de Pacientes com Infarto do Miocárdio com Supradesnivelamento do Segmento ST Submetidos à Intervenção Coronária Percutânea Primária
Source: Arq Bras Cardiol. 2023 Jun 1;120(6):e20220673. [Article in Portuguese] doi: 10.36660/abc.20220673 (PMC10263405; doi:10.36660/abc.20220673)
Supplement: Translation

# Translation: Sex Differences in Outcomes of ST Elevation Myocardial Infarction Patients Submitted to Primary Percutaneous Coronary Intervention

**Authors:** Victoria B. Milan, Yasmin F. S. Alves, Guilherme P. Machado, Gustavo Neves de Araujo, Ana Maria Krepsky, Angelo Chies, Matheus Niches, Julia Fracasso, Sandro Cadaval Goncalves, Marco Wainstein, Carisi Anne Polanczyk

**Affiliations:** 1 Brazil Universidade Federal de Ciências da Saúde de Porto Alegre Faculdade de Medicina, Porto Alegre, RS – Brazil; 2 Brazil Hospital de Clínicas de Porto Alegre, Porto Alegre, RS – Brazil; 3 Brazil Programa de Pós-Graduação em Cardiologia e Ciências Cardiovasculares, Universidade Federal do Rio Grande do Sul, Porto Alegre, RS – Brazil; 4 Brazil Instituto de Cardiologia de Santa Catarina, São José, SC – Brazil; 5 Brazil Imperial Hospital de Caridade, Florianópolis, SC – Brazil; 6 Brazil Universidade Federal do Rio Grande do Sul, Porto Alegre, RS – Brazil

**Keywords:** ST-elevation myocardial infarction (STEMI, sex characteristics, elderly, percutaneous coronary interventions

## Abstract

**Background:**

Several studies have shown that women are usually undertreated and have worse outcomes after ST-segment elevation myocardial infarction (STEMI), hence the need to investigate questions related to sex in Brazil to better deal with the problem.

**Objective:**

To determine whether female sex is still associated with adverse events in a contemporary cohort of patients with STEMI undergoing primary percutaneous coronary intervention (pPCI).

**Methods:**

This was a prospective cohort study of STEMI patients submitted to pPCI in a tertiary university hospital between March 2011 and December 2021. Patients were categorized into groups based on their sex at birth. The primary clinical outcome was long-term MACCE. Patients were followed-up for up to five years. All hypothesis tests had a two-sided significance level of 0.05.

**Results:**

Among 1457 patients admitted with STEMI in the study period, 1362 were included and 468 (34.4%) were women. Female patients had a higher prevalence of hypertension (73% vs. 60%, p <0.001), diabetes (32% vs. 25%, p=0.003) and Killip class 3-4 at hospital admission (17% vs. 12%, p=0.01); TIMI risk score was higher among women (4 [2, 6] vs. 3 [2, 5], p<0.001). In-hospital mortality was not different between groups (12.8% vs. 10.5%, p=0.20). In-hospital MACCE (16.0% vs. 12.6%, p=0.085) and long-term MACCE (28.7% vs. 24.4%, p=0.089) were numerically higher in women, with borderline significance. After multivariate analysis, female sex was not associated with MACCE (HR = 1.14; 95% CI 0.86 – 1.51; p = 0.36).

**Conclusion:**

In a prospective cohort of STEMI patients submitted to pPCI, female patients were older and had more comorbidities at baseline, but no significant differences were found in terms of long-term adverse outcomes.

## Introduction

Cardiovascular disease is among the main causes of morbidity and mortality worldwide.^1^ The prevalence of acute coronary artery disease (CAD) in Brazil has been increasing in both men and women, and already represents 13% of deaths in the overall population.^2^ Historically, men are more affected by CAD than women.^3^ Men experience the first myocardial infarction (MI) at least seven years before women.^3,4^ However, studies have shown that women, even with fewer events, have worse outcomes after an acute MI, especially those with ST-segment elevation (STEMI).^5,6^

Studies have reported several plausible socio-environmental theories for worse outcomes among women who present with MI.^7^ In addition, much of the current knowledge about sex differences in STEMI management is based on studies conducted in high-income countries. World statistics show that cardiovascular mortality rates in these countries have dropped around 10% in the last 20 years, while in lower-middle-income countries, such as Brazil, these rates have increased by around 40%.^1^ These same data also confirm that there are significant differences in healthy life expectancy between men and women from different economic and geographic situations.

Considering primary percutaneous coronary intervention (pPCI) the standard treatment in patients admitted with STEMI,^8^ this prospective cohort study aims to investigate the relationship between sex and adverse outcomes in patients admitted with STEMI submitted to primary PCI in a tertiary care hospital in southern Brazil.

## Methods

### Data, study design and population

This prospective study was conducted in a tertiary university hospital in Southern Brazil between March 2011 and December 2021. Patients eligible for inclusion were consecutive adults (≥ 18 years of age) with suspected STEMI, based on the presence of typical chest pain at rest associated with ST-segment elevation or abnormalities that met the diagnostic criteria for STEMI according to current guidelines.^8^ Exclusion criteria were non-ST elevation MI (NSTEMI), MI with non-obstructive coronary arteries, and other final diagnosis. Other details of our protocol have been described elsewhere.^9^ All patients provided written informed consent. This study was approved by the Institutional Research Ethics Committee. Manuscript writing was guided according to STROBE guideline for reporting observational studies.^10^

Blood samples were collected by venipuncture on admission for general laboratory testing. All patients were treated with optimal medical therapy according to current guidelines.^8^ PCI strategies (i.e., pre-dilation, direct stent placement, post-dilation) were performed at the operator’s discretion. Echocardiography was performed within 48 hours of admission according to hospital`s routine.

Data from medical records were transferred to standardized case report forms (CRFs). The following variables were collected: baseline clinical characteristics, medical history, procedure characteristics, reperfusion strategy, pharmacological treatment in intensive care unit, need for hemodynamics monitoring devices and discharge therapies. Killip classification was used at the first evaluation at clinical admission before coronary revascularization. Thirty-day and long-term follow-up was conducted by clinical visit and telephone contact to a maximum of 60 months. Study data were transferred and managed using REDCap electronic data capture tools hosted at Hospital de Clínicas de Porto Alegre. Patients were categorized by sex.

### Outcomes

The primary clinical outcome was long-term major adverse cardiac and cerebrovascular events (MACCE) – a composite outcome of all-cause mortality, new MI, stroke, stent thrombosis and target vessel revascularization). Treatment of non-culprit lesions was not considered as new revascularization. Secondary outcome was the individual analysis of MACCE. New MI was defined in accordance with the most recent universal definition of MI.^11^ A secondary analysis was performed for mortality in subgroups stratified by age. Stroke was defined as a new, sudden-onset focal neurologic deficit, of presumably cerebrovascular cause, irreversible (or resulting in death) and not caused by other readily identifiable causes.

Procedural outcomes were also described. Successful procedure was defined as final a thrombolysis in myocardial infarction (TIMI) score 2 or 3 flow and residual stenosis <30%. No reflow was defined as suboptimal myocardial reperfusion through a part of coronary circulation without angiographic evidence of mechanical vessel obstruction. Distal embolization was defined as a distal filling defect with an abrupt ‘cutoff’ in one of the peripheral coronary artery branches of the infarct-related vessel, distal to the site of angioplasty. Cardiac arrest was defined as cardiac arrest occurring during the procedure and requiring resuscitation procedures (i.e. ventilation, chest compression, defibrillation).

### Statistical analysis

Continuous variables were expressed as mean ± standard deviation (SD) or median (interquartile range), according to data normality. The normality of the distribution of each variable was assessed by the Shapiro-Wilk test. Categorical variables were expressed as relative and absolute frequencies. Patient groups were compared using independent samples Student’s t test (for normally distributed variable) or Mann-Whitney U test (for other variables) for continuous variables and χ2 test or Fisher’s exact tests for categorical variables. For multivariate analysis, a Cox regression analysis for primary outcome was performed and clinically important variables were included. Multivariate model was adjusted by sex, age, anterior wall MI, Killip class 3 or 4, hypertension, diabetes, creatinine at admission, multivessel disease, previous use of acetylsalicylic acid (ASA), previous MI, previous or current drugs, pain-to-door time, smoking, left ventricular ejection fraction (LVEF), angiographic success, body mass index, complete atrioventricular (AV) block. Kaplan-Meier survival curves were built to present the unadjusted time-to-event data for the investigated endpoints and were compared using the log-rank test using MedCalc Statistical Software version 14.8.1 (MedCalc Software bvba, Ostend, Belgium). All remaining statistical analyses were conducted SPSS for Windows, version 26.0. (IBM Corp., Armonk, NY). All hypothesis tests had a two-sided significance level of 0.05

## Results

### Baseline clinical characteristics

Of the 1457 patients admitted with STEMI in the study period, 1362 (468 female and 894 male) were included in the analysis (Figure 1).

**Figure 1:** – Flowchart of patient inclusion. STEMI: ST segment elevation myocardial infarction; NSTEMI: non-ST elevation myocardial infarction.

Mean age was 62.8 years in women and 60.2 years in men. Hypertension, diabetes, Killip class 3-4 at admission and complete AV block were more common in female patients. TIMI risk score was higher among women. Male patients had lower post-MI ejection fraction. Other baseline characteristics of female and male patients are summarized in Table 1.

**Table 1:** – Baseline characteristics of patients by sex

	Female (n=468)	Male (n=894)	p-value
Age, years	62.8 (±12.2)	60.2 (±11.5)	<0.0001
BMI, kg/m^2^	26 (23.3-29.2)	26.7 (24.2-29.8)	0.01
Caucasian	407 (87)	799 (89.6)	0.24
Hypertension	342 (73.1)	538 (60.2)	<0.001
Diabetes mellitus	152 (32.5)	223 (24.9)	0.003
Insulin therapy	54 (35.5)	56 (24.9)	0.02
Family History of CAD	74 (16.2)	165 (18.9)	0.20
Alcohol and/or drug use	15 (3.2)	142 (16.0)	<0.0001
Atrial Fibrillation	13 (2.8)	25 (2.8)	0.97
Previous or current smoking	290 (62.0)	588 (65.8)	0.16
Dyslipidemia	71 (15.2)	130 (14.6)	0.78
Aspirin use	87 (18.6)	148 (16.6)	0.36
Previous AMI	55 (11.8)	133 (14.9)	0.11
Previous stroke	47 (10.0)	68 (7.6)	0.12
Previous CABG	11 (2.4)	27 (3.0)	0.48
Previous PCI	52 (11.2)	104 (11.7)	0.77
CKD	19 (4.1)	46 (5.1)	0.37
Psychiatric disorder	42 (9.0)	41 (4.6)	0.001
On-hours	242 (51.7)	508 (56.8)	0.13
Weekends	161 (34.4)	285 (31.9)	0.35
**Killip Class**			0.003
I	284 (60.7)	630 (70.5)	
II	105 (22.4)	157 (17.6)	
III	21 (4.5)	26 (3.1)	
IV	58 (12.4)	79 (8.8)	
Killip III-IV	79 (16.9)	107 (12.0)	0.01
Complete AV block	40 (8.5)	42 (4.7)	0.005
SCA	45 (9.6)	93 (10.4)	0.64
**CAD Extension**			0.06
1 vessel	176 (37.8)	298 (33.7)	
2 vessels	124 (26.7)	289 (32.7)	
Multivessel Disease	165 (35.5)	298 (33.7)	
Thrombolysis	17 (3.7)	38 (4.3)	0.59
Complete Revascularization	121 (59.3)	221 (56.4)	0.49
Pain-to-Door, min	300 (180-503)	300 (180-480)	0.24
Door-to-Ballon, min	70 (57-95)	73 (60-98)	0.35
Total Ischemia time, min	365 (250-561)	370 (243 -548)	0.48
SBP, mmHg	130 (110-150)	130 (112-150)	0.48
HR, bpm	82 (70-94)	80 (69-93)	0.20
TIMI risk score	4 (2-6)	3 (2-5)	<0.0001
Creatinine, mg/dL	0.82 (0.68-1.1)	1.01 (0.85-1.29)	<0.0001
Hemoglobin, g/dL	12.7 (11.6-13.7)	14.2 (13-15.1)	<0.0001
NLR	6.61 (3.4-9.2)	6.5 (4.1-10.0)	0.053
LVEF, %	53 (41-62)	48 (40-58)	<0.0001
**Procedural Characteristics**			
Contrast, ml	152 (120-206)	175 (140-230)	0.012
Radiation, mGy	1282 (806-2246)	1649 (1026-2543)	<0.0001
Total Stents	1 (1-2)	1 (1-2)	0.41
Stent length, mm	32 (22-51)	30 (23-48)	0.90

Values are expressed as mean (SD standard deviation), median (interquartile range) or number (%); BMI: body mass index; AMI: acute myocardial infarction; CABG: coronary artery bypass grafting;PCI: percutaneous coronary intervention; CKD: chronic kidney disease; AV block: Atrioventricular block; SCA: Sudden cardiac arrest, CAD coronary artery disease, D2B door to balloon; SBP: systolic blood pressure; HR: heart rate; NLR: neutrophil-to-lymphocyte ratio; LVEF: left ventricular ejection fraction. Source: author.

### Outcomes

The incidence of long-term (median of 41 months) MACCE was 31.4% in female and 26.5% in males (hazard ratio (HR) = 1.14; 95% confidence interval (95% CI) = 0.86–1.51; p =0.36) (Figure 2 and central illustration).

**Figure 2:** – Time-to-event curves for the primary outcome major adverse cardiac and cerebrovascular events MACCE. Event rates were calculated using the Kaplan–Meier method and compared with the use of the log-rank test. Source: author

**Central Illustration:** : Sex Differences in Outcomes of ST Elevation Myocardial Infarction Patients Submitted to Primary Percutaneous Coronary Intervention

Overall, in-hospital mortality was 11.3%, with no difference between female and male patients. (Figure 3).

**Figure 3:** – Incidence of mortality by sex and age groups.

Incomplete revascularization at discharge was associated with increased mortality among male (14 vs. 6.3%, p=0.01), but not female patients (10.7 vs. 12%, p=0.82). No differences were observed in in-hospital MI, stroke and MACCE, or in MI, stroke, and target vessel revascularization at long term between the groups (Table 3).

**Table 3:** – Adverse outcomes according to born sex

	Female (n=468)	Male (n=894)	p-value
**Primary Outcome**			
Long Term MACCE	148 (31.7)	238 (26.7)	0.36
**Secondary Outcomes**			
In-Hospital Death	60 (12.8)	94 (10.5)	0.20
In-Hospital New AMI	10 (2.1)	14 (1.6)	0.44
In-Hospital Stent thrombosis	5 (1.1)	10 (1.1)	0.93
In-Hospital Stroke	10 (2.1)	11 (1.2)	0.19
In-hospital MACCE	75 (16.0)	113 (12.6)	0.08
**Long-Term**			
Death	88 (18.8)	142 (15.9)	0.17
AMI	38 (8.2)	58 (6.5)	0.25
Stroke	23 (4.9)	26 (2.9)	0.05
Stent Thrombosis	22 (4.7)	38 (4.3)	0.70
Target Vessel Revascularization	34 (7.3)	52 (5.8)	0.29

AMI: acute myocardial infarction; MACCE: major adverse cardiac events. Source: author

Medications at hospital discharge were different between female and male patients. While aldosterone receptor blockers and calcium channel blockers were prescribed more commonly to female patients, ACE inhibitors were more common in male patients’ discharge list. No differences were found for the other medications (Table 4).

**Table 4:** – Medications at hospital discharge among male and female

	Female (n=468)	Male (n=894)	p-value
Aspirin	381 (93.4)	755 (94.4)	0.49
Clopidogrel	374 (91.7)	739 (92.4)	0.65
Statin	368 (90.2)	729 (91.1)	0.59
Beta-blocker	355 (87.0)	710 (88.8)	0.37
Other antiplatelet	5 (1.2)	12 (1.6)	0.58
ARB	27 (6.6)	29 (3.6)	0.01
ACEI	312 (76.5)	660 (82.5)	0.01
Spironolactone	27 (6.6)	66 (8.3)	0.31
Digoxin	11 (2.7)	20 (2.5)	0.83
Warfarin	18 (4.4)	42 (5.3)	0.52
NOAC	7 (1.7)	28 (3.5)	0.08
Calcium Channel Blocker	16 (3.9)	16 (2.0)	0.04

BRA: angiotensin receptor blockers; ACEI: Angiotensin-converting enzyme inhibitors; NOAC: Non-vitamin K antagonist oral anticoagulants; Source: author.

### Multivariate analysis

Age, Killip 3-4 at admission, multivessel disease, previous use of ASA, and LVEF were independent predictors of MACCE in the overall population. Creatinine and LVEF were independent predictors of MACCE in female population but not in males. Anterior wall MI was predictor of MACCE in men but not in women (Table 5).

**Table 5:** – Predictors of MACCE in overall patients and according to sex

	Overall				Female				Male			
		
	HR	95% CI		p-value	HR	95% CI		p-value	HR	95% CI		p-value
Female	1.14	0.86	1.51	0.36								
Age	1.02	1.00	1.03	0.003	1.00	0.98	1.02	0.66	1.16	0.82	1.64	0.38
Anterior Wall MI	1.09	0.83	1.44	0.52	0.72	0.44	1.19	0.20	1.92	1.19	3.08	<0.01
Killip 3 or 4	2.15	1.53	3.03	<0.01	1.63	0.92	2.89	0.09	0.86	0.61	1.23	0.43
Creatinine	1.10	0.99	1.22	0.05	1.57	1.23	1.99	<0.01	1.31	0.88	1.93	0.17
Multivessel Disease	1.65	1.21	2.26	<0.01	2.66	1.51	4.67	<0.01	1.73	1.12	2.67	0.01
ASA previous use	1.59	1.14	2.23	0.01	1.01	0.57	1.78	0.96	0.89	0.54	1.46	0.65
LVEF	0.98	0.97	0.99	0.01	0.97	0.95	0.99	<0.01	0.71	0.39	1.28	0.26

ASA: acetylsalicylic acid; LVEF: left ventricular ejection fraction; MI: myocardial infarction; CI: confidence interval.

The model was adjusted by sex, age, anterior wall MI, Killip class 3 or 4, hypertension, diabetes, creatinine at admission, multivessel disease, ASA previous use, previous MI, previous or current drug use, pain-to-door, smoking, LVEF, angiographic success, body mass index, complete AV block.

## Discussion

In a prospective cohort of STEMI patients undergoing pPCI, we found no significant differences between female and male genders in terms of mortality and MACCE. Female patients had higher risk characteristics at baseline and numerically higher in-hospital and long-term adverse outcomes, although differences were partially neutralized after multivariate analysis. Finally, women were less likely to be discharged with guideline-oriented medical therapy. This study reproduces findings of previous works and contributes to the limited data on sex differences in STEMI management in developing countries.

As virtually all studies published in STEMI, the prevalence of male patients was higher in this cohort and can be explained by two aspects. First, men have a higher prevalence of acute coronary syndromes compared to women (2.3% vs 1.2%),^2^ and the present analysis found that the prevalence of previous AMI was higher in men. Second, women are underdiagnosed due to atypical symptom and have lower access to pPCI, with several studies indicating that men are almost twice as likely to undergo any reperfusion therapy.^12-15^

Differences in demographic profile between sexes were also similar with previous studies, in which women are usually older and have more comorbidities.^13-15^ Regarding the age factor, estrogen is thought to have a protective effect,^7,15^and women seem to have a greater adherence to primary prevention.^3^ A previous analysis with 1.2 million patients with STEMI revealed higher mortality in women aged 19-49 years in a model adjusted for reperfusion therapy (3.9% vs 2.6%, p=0.003), which, however, was not confirmed in our study.^14^ Comorbidities exert a different weight in the pathogenesis of AMI between genders, impacting more women. Diabetes mellitus, for example, increases the chances of AMI by up to three times in the female population when compared to the male population.^12^ In our analysis, different variables were independent predictors of long-term MACCE among female (creatinine and LVEF) and male patients (anterior wall MI).

In our study, significant baseline differences between men and women were not translated into statistically significant differences in in-hospital mortality and MACCE, but studies with different populations have shown conflicting results. A study evaluating 2.8 million patients with STEMI undergoing pPCI found significantly higher rates of mortality and vascular complications among women.^16^ Spanish researchers analyzed a sample of 680 nonagenarians, with an incidence of 45% of STEMI and 35% of PCI, and concluded that in-hospital mortality rates were no different between men and women (16% vs. 18%; p = 0,4).^16^ An Australian study investigated pre- and post-procedure revascularization status in STEMI patients undergoing PCI, and concluded that the mortality of female gender is strongly associated with incomplete revascularization.^6^ In the present analysis, this finding was present in male patients.

Current guideline-directed management and therapies in cardiovascular disease are based on data obtained predominantly from male patients.^12^ Recently, several initiatives have proposed a greater attention on achieving gender equity in cardiovascular science, to improve our understanding in terms of pathophysiology of cardiovascular disease and their impact in adverse outcomes.^17,18^Therefore, studies on these issues in different scenarios remain important to improve our daily clinical practice.

This study has limitations that are inherent to observational studies. Some data were obtained retrospectively and others through telephone calls, which may determine less reliable information. In addition, there are limitations due to the relatively small sample size compared to larger populational studies and short follow-up time. This was a single center study in southern Brazil and may not be representative of all the country that has significant cultural differences across regions. Moreover, we found a high mortality rate in our sample, which may be justified by high disease severity of our patients (approximately 13% were Killip class III/IV), possibly due to late presentation, representing a very high baseline risk. However, this study is a registry of consecutive and unselected patients from a tertiary referral hospital for the treatment of acute coronary syndromes, so the data shown are highly applicable in daily clinical practice.

## Conclusion

In a contemporary prospective cohort of STEMI patients submitted to pPCI, female patients were older and had more comorbidities at baseline, but no significant differences were found in terms of in-hospital and long-term adverse outcomes. Unfortunately, women were less likely to be discharged with guideline-oriented medical therapy. We hope this information helps physicians to provide better care for this group of patients in different social contexts.
